# Metabolomic profiling of postmortem aged muscle in Japanese Brown beef cattle revealed an interbreed difference from Japanese Black beef

**DOI:** 10.5713/ab.22.0202

**Published:** 2022-09-02

**Authors:** Susumu Muroya, Riko Nomura, Hirotaka Nagai, Koichi Ojima, Kazutsugu Matsukawa

**Affiliations:** 1 Animal Products Research Division, NARO Institute of Livestock and Grassland Science, Tsukuba, Ibaraki 305-0901, Japan; 2 Kochi Prefectural Seibu Animal Hygiene Service Center, Shimanto, Kochi 787-0019, Japan; 3 Department of Agriculture and Marine Science, Kochi University, Nankoku, Kochi 783-8502, Japan

**Keywords:** Beef, Capillary-Electrophoresis Time-of-Flight Mass Spectrometry (CE-TOFMS), Japanese Brown, Metabolomics, Postmortem Aging

## Abstract

**Objective:**

Japanese Brown (JBR) cattle, especially the Kochi (Tosa) pedigree (JBRT), is a local breed of moderately marbled beef. Despite the increasing demand, the interbreed differences in muscle metabolites from the highly marbled Japanese Black (JBL) beef remain poorly understood. We aimed to determine flavor-related metabolites and postmortem metabolisms characteristic to JBRT beef in comparison with JBL beef.

**Methods:**

Lean portions of the *longissimus thoracis* (loin) muscle from four JBRT cattle were collected at 0, 1, and 14 d postmortem. The muscle metabolomic profiles were analyzed using capillary electrophoresis time-of-flight mass spectrometry. The difference in postmortem metabolisms and aged muscle metabolites were analyzed by statistical and bioinformatic analyses between JBRT (n = 12) and JBL cattle (n = 6).

**Results:**

A total of 240 metabolite annotations were obtained from the detected signals of the JBRT muscle samples. Principal component analysis separated the beef samples into three different aging point groups. According to metabolite set enrichment analysis, postmortem metabolic changes were associated with the metabolism of pyrimidine, nicotinate and nicotinamide, purine, pyruvate, thiamine, amino sugar, and fatty acid; citric acid cycle; and pentose phosphate pathway as well as various amino acids and mitochondrial fatty acid metabolism. The aged JBRT beef showed higher ultimate pH and lower lactate content than aged JBL beef, suggesting the lower glycolytic activity in postmortem JBRT muscle. JBRT beef was distinguished from JBL beef by significantly different compounds, including choline, amino acids, uridine monophosphate, inosine 5′-monophosphate, fructose 1,6-diphosphate, and betaine, suggesting interbreed differences in the accumulation of nucleotide monophosphate, glutathione metabolism, and phospholipid metabolism.

**Conclusion:**

Glycolysis, purine metabolism, fatty acid catabolism, and protein degradation were the most common pathways in beef during postmortem aging. The differentially expressed metabolites and the relevant metabolisms in JBRT beef may contribute to the development of a characteristic flavor.

## INTRODUCTION

Japanese Brown (JBR) cattle, compared to the Japanese Black (JBL) breed, are a minor population breed with moderately marbled beef and high growth performance [1]. In 2021, approximately 22,400 animals were reared in Japan. To date, the genetic background [2], growth performance [3], carcass [4,5] and beef characteristics of JBR cattle have been investigated [6,7]. This breed comprises two pedigrees, 10% animals of which are the Kochi (Tosa; JBRT), and the remainder are the Kumamoto [8]. In recent years, JBR beef has been accepted by health conscious consumers owing to a balanced composition of lean muscle and intramuscular fat [9]. Despite the increasing demand for this beef in the market, the details of its skeletal muscle properties remain poorly understood. The crude fat content in loin muscle was 26.6% on average (n = 45) in another JBR cattle (Kumamoto pedigree), whereas the content was 39.7% in JBL cattle, the original Wagyu breed with highly marbled beef [1]. In addition, levels of carnosine, taurine, glutamine, and alanine were 9.63, 6.70, 5.21, and 3.71 μmol/g, respectively, in the loin of JBR Kumamoto cattle, whereas those in JBL cattle were 6.90, 5.33, 3.64, and 2.95 μmol/g, respectively. Total peptide content in the JBR and JBL cattle was 4.78 and 2.49 mg/g, respectively [1]. These results suggest that the levels of flavor-related metabolites in the loin muscle are higher in JBR cattle than in JBL cattle.

In farm animals, metabolites play physiologically essential roles in skeletal muscles and contribute to nutritional value and palatability after death [10]. During postmortem aging of porcine and bovine meat, proteins, glycogen, sugars, and energy-charged metabolites, such as adenosine triphosphate (ATP), are degraded through biochemical reactions [11–18]. An appropriate process of postmortem meat aging leads to preferable meat flavor development by increasing metabolites, not only amino acids (AAs) but also nucleotide degradation products such as inosine 5′-monophosphate (IMP) as flavor and taste components [11,19,20]. In addition, these metabolites contribute to formation of “meaty aroma” as precursors of the flavor-related compounds [21]. In particular, free AAs and peptides react with sugar-related metabolites via the Maillard reaction in meat during cooking [22]. These compounds can be further converted via autooxidation and evaporated as volatile flavor compounds, such as 2,5-dimethyl-4-hydroxy-3(2H)-furanone (furaneol) [23]. Thus, JBR beef may have a characteristic meat flavor that is stronger than that of JBL beef.

To understand the mechanisms underlying metabolite changes in such processes and conditions of meat production, metabolomic approaches have been employed in studies on postmortem meat aging [12,24–29]. Recently, we demonstrated that postmortem metabolic changes progress in the *longissimus thoracis* (LT) muscle metabolism of JBL cattle, including protein digestion, glycolysis, citric acid cycle, pentose phosphate pathway, and the metabolism of nicotinamide, purine, and glutathione [24]. In addition, metabolomic profiles are affected by the genetic background of the breed in animals, and therefore, it is expected that metabolomic approaches can identify breed-specific muscle metabolites that characterize the meat [30,31]. Taking the interbreed difference into account, this led us to hypothesize that, in JBR cattle, skeletal muscle metabolism and the metabolites are different from those in JBL cattle. However, to date, interbreed differences in the metabolomes of bovine skeletal muscle and beef products have been poorly addressed. In particular, little is known about JBR muscle metabolites and their differences from those of JBL cattle, which may have an impact on interbreed differences in beef flavor.

To address this, we aimed to capture the metabolome profiles in the lean portion of LT muscle in JBR cattle to characterize the beef, especially focusing on postmortem aging, using capillary-electrophoresis time-of-flight mass spectrometry (CE-TOFMS) metabolomics targeting water-soluble metabolites. The metabolomic data of JBR cattle were subjected to bioinformatic analyses, including metabolite pathway analysis. The data were compared to a dataset of postmortem muscle aging in JBL cattle. Interbreed differences are discussed in this article.

## MATERIALS AND METHODS

### Animals and muscle samples

The animals were reared as outlined in the Guide for the Care and Use of Experimental Animals for JBRT cattle (Animal Care Committee of Kochi University) and JBL cattle (Animal Care Committee of the NARO Institute of Livestock and Grassland Science [NILGS]), and the committees approved the study (approval number 1611C010). Individual diets were designed for JBRT cattle to satisfy energy requirements and other nutrients based on the standard diet model in the Japanese Feeding Standard for Beef Cattle (JFSBC, 2008 ed.) [32]. The dietary composition was the same as that of a previous JBL cattle experiment [24]. According to the JFSBC, the recommended levels of dry matter, neutral detergent fiber, total digestible nutrients, and crude protein in diet are 87.1%, 33.5%, 75.8%, and 11.8%, respectively (all values are expressed as a percentage of total mixed rations).

All efforts were made to minimize animal suffering. To analyze the effect of postmortem aging, the loin LT muscle blocks were excised, and a portion of the LT muscles was dissected from 28 month-aged two JBRT steers and two heifers (584–774 kg), and three JBL (632–739 kg) steers within 1 h after slaughter at a commercial abattoir or the slaughterhouse of the NILGS. To avoid contamination of intramuscular fat, only a lean portion of small muscle pieces was carefully collected from four or five locations in the core part of each LT muscle at time 0 postmortem (D0). After overnight storage of the loin cut block at 2°C, from the remaining block, small lean muscle portions were dissected at 24 h (D1), and after continuous storage of the LT muscles in a vacuumed bag at 2°C, further muscle pieces were picked up at 336 h (D14) postmortem.

For the comparative analysis of aged beef samples (D14) between cattle breeds, 12 JBRT (six heifers and six steers) and six JBL steer beef samples after 14-day aging were obtained from the NILGS slaughterhouse or a commercial abattoir. To minimize the deterioration of the metabolites, mincing of the muscle samples was avoided. All subsequent small pieces of lean muscle were collected, frozen in liquid nitrogen, and stored at −80°C until use. Muscle pH was measured according to a previously described method [12].

In this study, (1) changes during postmortem aging in JBRT beef between the time points using 240 metabolites (relatively quantified), (2) changes during postmortem aging in JBRT and JBL between the breeds using 110 metabolites (absolutely quantified), and (3) metabolome profile of 14-day aged JBRT and JBL beef between the breeds using 110 metabolites (absolutely quantified) were compared. The samples available for each comparison were limited to (1) two steers and two heifers of JBRT, (2) two steers and two heifers of JBRT and three JBL steers, and (3) six steers and six heifers of JBRT and six JBL steers, respectively. We used all the available samples meeting each comparison mentioned above.

### Metabolomic measurement by CE-TOFMS

Samples for CE-TOFMS measurements were prepared as previously described [24]. In brief, the frozen muscle pieces (50.0 to 100.0 mg) were immediately soaked in 50% acetonitrile containing 10 μM Internal Standard Solution 1 (Human Metabolome Technologies, Tsuruoka, Japan), followed by homogenization at 0°C. After centrifugation at 2,300×*g* for 5 min at 4°C, the upper layer solution was filtered through a 5-kDa cutoff membrane. The filtrate was lyophilized, suspended in Milli-Q water, and analyzed using CE-TOFMS. Metabolomic measurements were performed using an Agilent CE capillary electrophoresis system (Agilent Technologies, Waldbronn, Germany). The analytical conditions were the same as those used in a previous study [24]. Briefly, cationic, and anionic metabolites were analyzed using a fused silica capillary (50 μm i.d. ×80 cm total length), with commercial cation and anion electrophoresis buffers (HMT Sheath Liquid: H3301-1001 and H3302-1021; Human Metabolome Technologies, Japan), respectively. The sample was injected at a pressure of 50 mbar for 10 s to detect cationic and anionic metabolites, with applied voltage at 30 kV. Electrospray ionization-mass spectrometry (ESI-MS) was conducted in the positive and the negative ion mode with the capillary voltage set at 4,000 and 3,500 V for the cationic and anionic mode analyses, respectively. The spectrometer scanned from 50 to 1,000 *m/z*.

### Data analysis of CE-TOFMS results

Raw data obtained by CE-TOFMS were processed using MasterHands (ver.2.18.0.1), as described in our previous study [24]. Briefly, Signal peaks corresponding to isotopomers, adduct ions, and other product ions of known metabolites were excluded, and all signal peaks potentially corresponding to authentic compounds were extracted, and then their migration times (MTs) were normalized using those of the internal standards. Thereafter, the alignment of peaks was performed according to the m/z values and normalized MT values. The compounds annotated in the Human Metabolome Database (HMD; ver. 4.0, http://www.hmdb.ca/) or the Kyoto encyclopedia of genes and genomes database (KEGG; http://www.genome.jp/kegg/) were further analyzed. The relative amounts of the annotated compounds over time were determined by comparing the peaks of compounds with the same MS properties. To compare the relative amounts of the compounds between time points, the peak areas were normalized to those of the internal standards and by sample weight. Then the relative levels of the compounds were compared within each metabolite between the aging time points. The absolute concentration of major metabolites, such as glycolytic products, AAs, and ATP degradation products, were determined using commercially available standards. In the comparative analysis, the abundance of undetected compounds was set to 0. The raw MS data file conversion, peak picking, noise reduction, and data alignment for multiple samples were conducted as previously described [24].

### Statistical analyses

To compare pH changes between the breeds, the data of JBRT (n = 4) and JBL (n = 3) were tested at each time point by Student’s *t*-test. The data were considered significantly different if p<0.05. Normalized relative content values were used for data analyses. MetaboAnalyst (https://www.metaboanalyst.ca/), a bioinformatics database for metabolomics, was used for principal component analysis (PCA), hierarchical cluster analysis (HCA), partial least squares-discriminant analysis (PLS-DA), analysis of variance (ANOVA), post-hoc multiple comparison tests, and metabolite set enrichment analysis (MSEA). PCA is an unsupervised method that allows unbiased identification of patterns of metabolites between samples. PLS-DA was used as a supervised multivariate statistical method to identify metabolites that differed between groups to account for unwanted biological variation between animals in PCs when using PCA. Metabolites with high variable importance in projection (VIP) are considered influential contributors to the discrimination model of PLS-DA. For the statistical analysis of postmortem JBRT beef aging, data were analyzed using two-way ANOVA with postmortem storage time and sex as the fixed effects, the individual animal as the random effect, and considering storage time as a within-subject factor. The comparison between JBRT (n = 12) and JBL (n = 6) beef metabolome profiles of the absolutely quantified compound data were analyzed using the Student’s *t*-test. Data were considered significantly different if the false discovery rate (FDR) was <0.05. The pH value of the JBRT beef was also analyzed by ANOVA, followed by a post-hoc multiple comparison test with the least significant difference. Data were considered significantly different at p<0.05.

## RESULTS

### Postmortem changes in *longissimus thoracis* muscle metabolome of JBRT cattle

**Figure 1:**
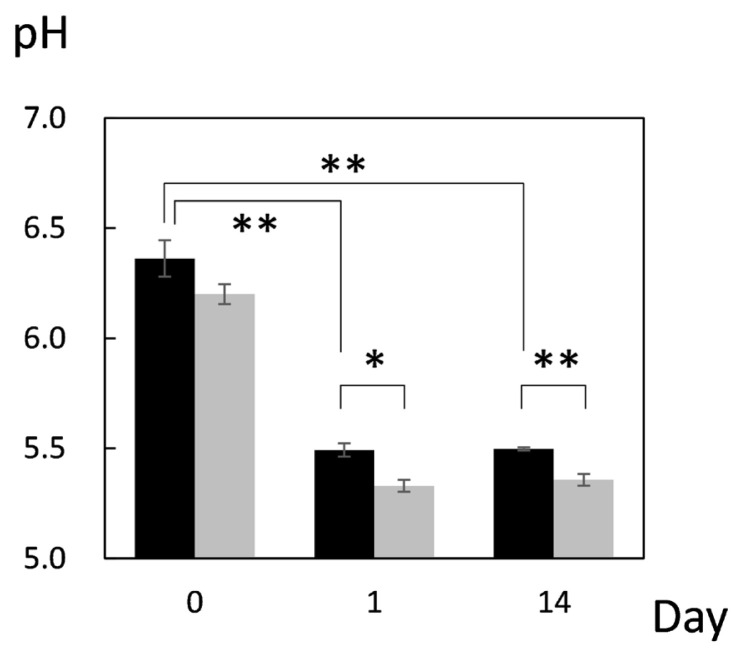
Postmortem pH decline in the *longissimus thoracis* (LT) muscle of Japanese Brown (black column) and Japanese Black (gray column) cattle. * and ** indicate significant difference between the breeds at p<0.05 and <0.01, respectively. Error bars indicate standard error of the mean.

First, pH of JBRT muscle was measured throughout postmortem aging period to ensure no abnormality in the pH decline. The pH values on postmortem days 0, 1, and 14 were 6.36, 5.49, and 5.49, respectively, indicating no aberrant metabolism in the postmortem beef samples (Figure 1). This result showed that the pH significantly declined from day 0 to day 1 (p<0.001), and thereafter was retained at day 14, with no change between days 1 and 14 (p≥0.05). A difference in pH between heifers (n = 2) and steers (n = 2) in JBRT breed was not observed throughout the aging period (p = 0.548). This pattern of pH decline is similar to that observed in JBL cattle previously reported [24], however, pH of JBRT muscle at day 1 and 14 postmortem was significantly higher than that of JBL steers (p<0.05) (Figure 1).

**Figure 2:**
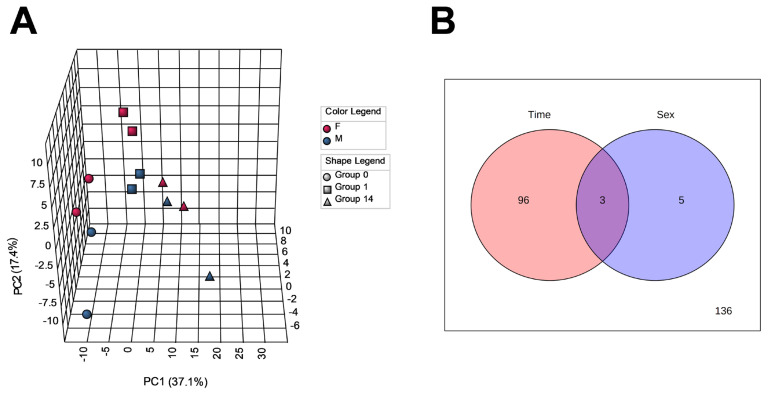
Results of principal component analysis (PCA, panel A) and analysis of variance (ANOVA, panel B) of metabolomic profiles during postmortem aging in Japanese Brown *longissimus thoracis* (LT) muscle. In panel A, the circle, square, and triangle symbols indicate LT samples taken at 0 (D0), 24 h (D1), and 168 h (D14) postmortem, respectively. Red (F), heifer; blue (M), steer.

To capture changes in the metabolome profiles during postmortem muscle aging of JBRT cattle, we measured the levels of the LT muscle metabolites of the heifers (n = 2) and steers (n = 2) at slaughter and postmortem day 1 and 14 samples by CE-TOFMS. As a result, 257 MS peaks were annotated across beef samples stored for 14 days. To characterize the LT muscle metabolome profiles based on metabolite distribution, PCA was performed using 240 annotated metabolites that are registered in HMD and/or KEGG (Figure 2A). The results showed that the beef metabolome profiles could be explained by the first two PCs: PC1 and PC2. The cumulative proportions of PC1, PC2, and PC3 were 37.1%, 17.4%, and 11.1%, respectively. The plot patterns of the beef samples on D0, D1, and D14 revealed a clear association between PC1 and postmortem muscle aging. In particular, samples D0 and D14 were segregated from each other by PC1, with the intermediate D1 samples in a transient state. PCA results indicated that the beef samples were not significantly distinguished by sex. According to the ANOVA results, 99 and 8 metabolite levels were significantly changed depending on the aging time and sex, respectively (FDR<0.05, Figure 2B), of which three metabolites were changed by both factors. No interaction was observed between aging time and sex.

The loading of 1,5-gluconolactone, inosine, cytidine, xanthine, uridine, 3-hydroxy-2-methyl-4-pyrone, *N*-acetylglucosamine-1/6-phosphate, gluconic acid, sedoheptulose-7-phosphate, and thiamine was highly positive for PC1 (Table 1). Samples D0 and D14 were plotted in the negative and positive areas of PC1, respectively, indicating that the JBRT beef samples on day 14 postmortem were characterized by an abundance of the above-mentioned compounds, such as inosine and cytidine. In addition, PC1 was negatively associated with the oxidized forms of nicotinamide adenine dinucleotide (NAD+), glycerol 3-phosphate (G3P), nicotinamide adenine dinucleotide phosphate (NADP+), guanosine triphosphate (GTP), phosphoribosyl diphosphate (PRPP), uridine triphosphate (UTP), ATP, oxidized glutathione (GSSG), *S*-lactoylglutathione, and malic acid, indicating that these compounds were abundant in the muscle at slaughter, but later decreased. In contrast, samples were not grouped by PC2 or PC3.

**Figure 3:**
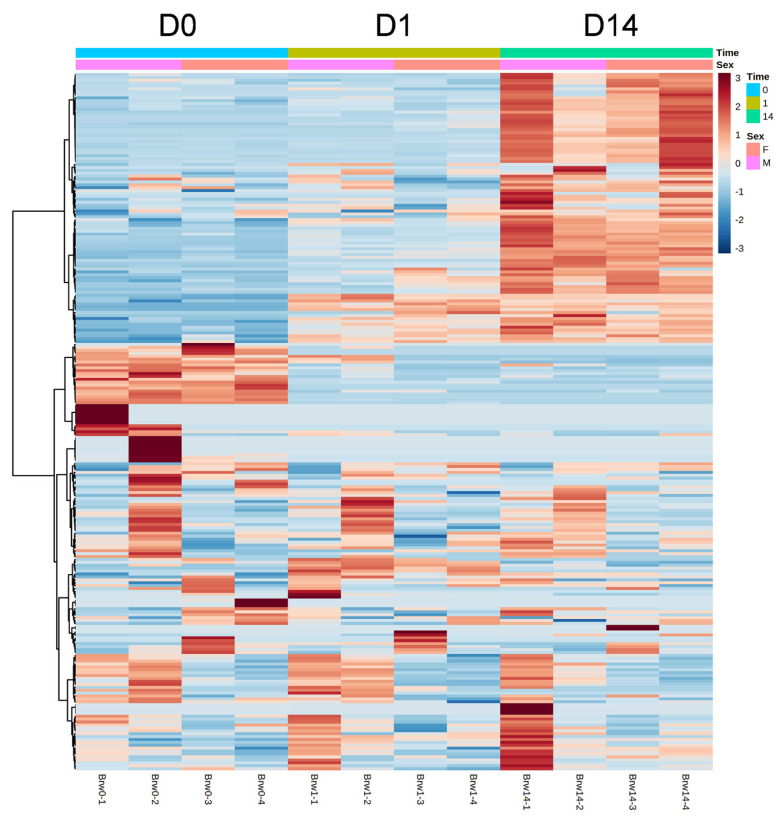
Heatmap result of hierarchical cluster analysis (HCA) of metabolomic changes during postmortem aging in the *longissimus thoracis* (LT) muscle of Japanese Brown cattle. The four cattle samples indicated under the graphic were allocated to each group at a specific time point (day 0, D0; day 1, D1; day 14, D14). The row displays the metabolite, and the column represents the sample. Metabolites with relatively low and high levels are displayed in light blue and brown, respectively. The brightness of each color corresponds to the magnitude of the difference when compared to the average value. F, heifer; M, steer.

**Figure 4:**
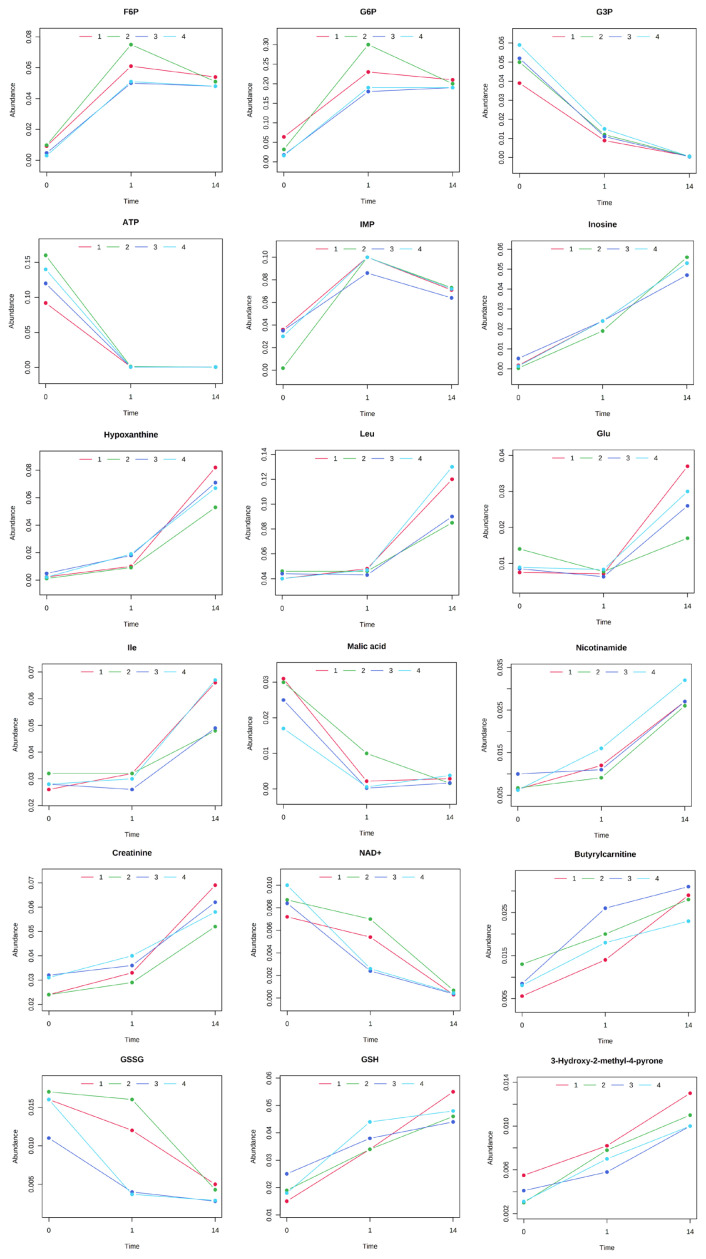
Postmortem changes in metabolite levels in the *longissimus thoracis* (LT) muscle of Japanese Brown cattle. The levels (vertical axis) are indicated in nmol/g. The horizontal axis indicates postmortem time (day 0, day 1, day 14).

The results of HCA using the above 240 annotated metabolites also revealed that the pattern of changes in metabolomes clearly depended on the post-mortem aging period (Figure 3). The metabolomic composition of water-soluble beef compounds was separated into several categories according to the pattern of changes over time. Of the significantly changed metabolites, glycolytic products such as fructose 6-phosphate (F6P) and glucose 6-phosphate (G6P) were increased by day 1 with no change afterward, whereas G3P was decreased, in a relative level within each metabolite in Figure 4. A purine metabolite generated by ATP degradation, IMP, increased within 24 h postmortem and thereafter slightly decreased, whereas inosine and hypoxanthine increased during aging over time. AAs, such as leucine, glutamate, and isoleucine, showed small changes within 24 h postmortem and then increased. Other metabolites, nicotinamide, creatinine, and butyrylcarnitine, were increased during aging, whereas malic acid, NAD+, and GSSG were decreased (Figure 4).

**Figure 5:**
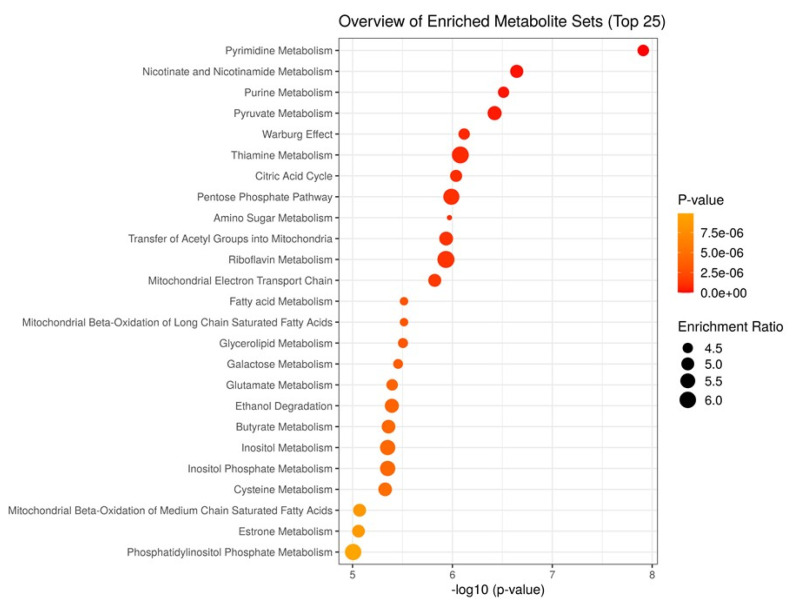
Result of metabolite set enrichment analysis for postmortem metabolite changes (day 0 [D0] versus day 14 [D14]) in the *longissimus thoracis* (LT) muscle of Japanese Brown cattle. Enrichment ratio is computed by hits/expected, where hits = observed hits; expected = expected hits.

To characterize the metabolism activated in the aged beef, we performed MSEA for JBRT beef using the annotated metabolites during postmortem aging. As the beef samples were not significantly classified into heifers or steers in PCA, we considered that the samples could be analyzed simply by the effect of postmortem aging time without the effect of sex. Potentially activated metabolites were extracted by comparing the metabolite distribution between D0 and D14 beef samples using MSEA (Figure 5). The results revealed that metabolism of pyrimidine, nicotinate and nicotinamide, purine, pyruvate, thiamine, and amino sugar; the Warburg effect; citric acid cycle; pentose phosphate pathway; and transfer of acetyl groups into mitochondria were predicted to be active in postmortem beef aging under the storage conditions (FDR <0.001), as well as other metabolic processes, including metabolism of fatty acid, butyrate, inositol, and glycerolipid.

### Interbreed differences in beef aging between JBRT and JBL cattle

**Figure 6:**
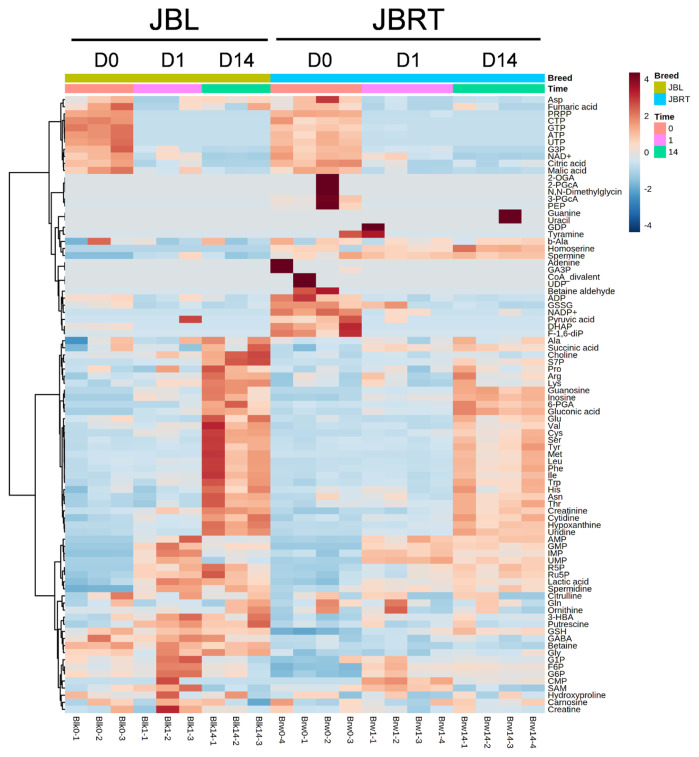
Heatmap result of hierarchical cluster analysis (HCA) of metabolomic changes during postmortem aging in the *longissimus thoracis* (LT) muscle of Japanese Black (JBL) and Japanese Brown (JBRT) cattle. The three JBL and four JBRT samples at a specific time point (day 0, D0; day 1, D1; day 14, D14) indicated under the heatmap were allocated to each breed group. The row displays the metabolite, and the column represents the sample. Metabolites with relatively low and high levels are displayed in light blue and brown respectively. The brightness of each color corresponds to the magnitude of the difference when compared to the average value.

**Figure 7:**
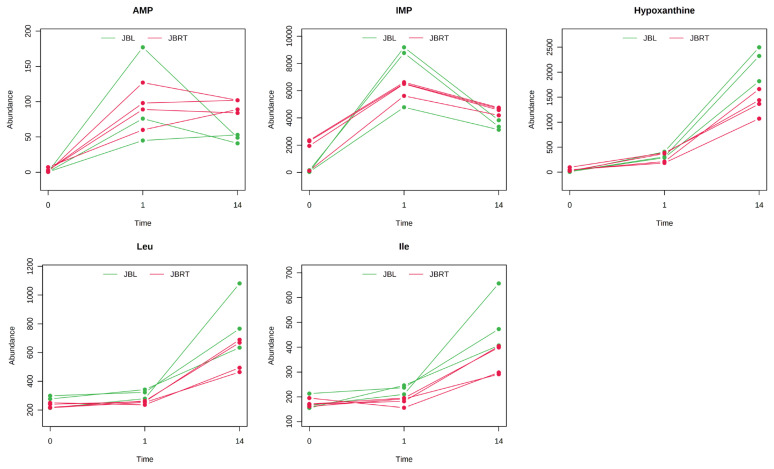
Postmortem changes in metabolite levels in the *longissimus thoracis* (LT) muscle of Japanese Black and Japanese Brown cattle. The levels (vertical axis) are indicated in nmol/g. The horizontal axis indicates postmortem time (day 0, day 1, day 14). Amino acids (AAs) are indicated by their three letter representations.

Metabolite generation and related metabolism in postmortem beef aging may differ between breeds of cattle. Accordingly, we aimed to identify the metabolites and metabolic pathways that differ between JBRT- and JBL-aged beef. To address this, the beef metabolome profiles of the two breeds in postmortem aging were compared using 110 absolutely quantified metabolite profiles. The lactate concentrations in JBRT beef were 81,641 and 89,340 nmol/g at day 1 and 14 postmortem, respectively, which were lower than those in JBL beef (115,982 and 105,429 nmol/g) at p<0.05 and p<0.10, respectively [24]. According to the HCA using postmortem aging samples of JBRT (n = 4) and JBL (n = 3) cattle, the beef samples of both breeds showed a trend of aging-dependent metabolite changes but also a slightly different pattern of the changes between the breeds (Figure 6). Some of the metabolites differed between JBRT and JBL beef during postmortem aging, as did the metabolites associated with purine metabolism, including adenine monophosphate (AMP), IMP, hypoxanthine, and AA metabolism, including leucine and isoleucine (Figure 7). Regarding metabolite changes, AAs generation and ATP degradation into hypoxanthine were relatively slow in JBRT beef compared to those in JBL beef.

**Figure 8:**
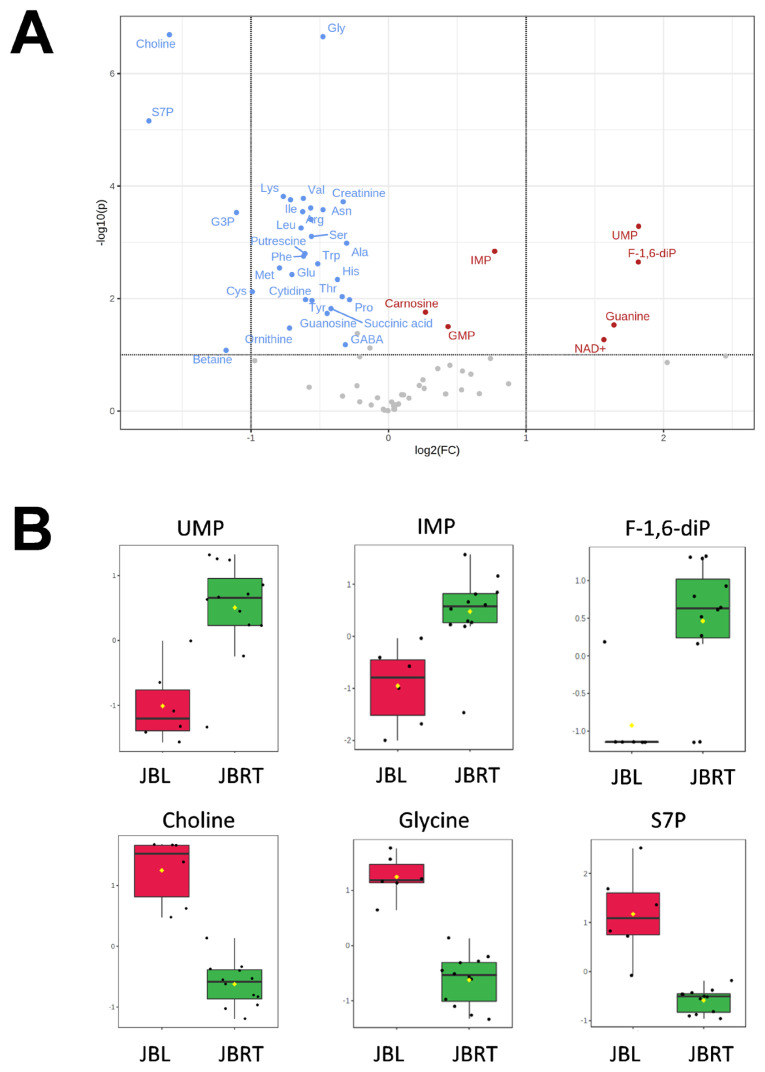
Differences in metabolite levels between Japanese black (JBL) and Japanese Brown (JBRT) *longissimus thoracis* (LT) muscles at day 14 postmortem. (A) Volcano plot. Horizontal axis indicates log_2_ fold change (JBRT/JBL ratio) for each metabolite, and vertical axis indicates −log10(*P*-value). Red, increased in JBRT; blue, decreased in JBRT. (B) Relative levels of metabolites in JBL and JBRT beef samples. Vertical axis indicates relative level normalized by autoscaling.

**Figure 9:**
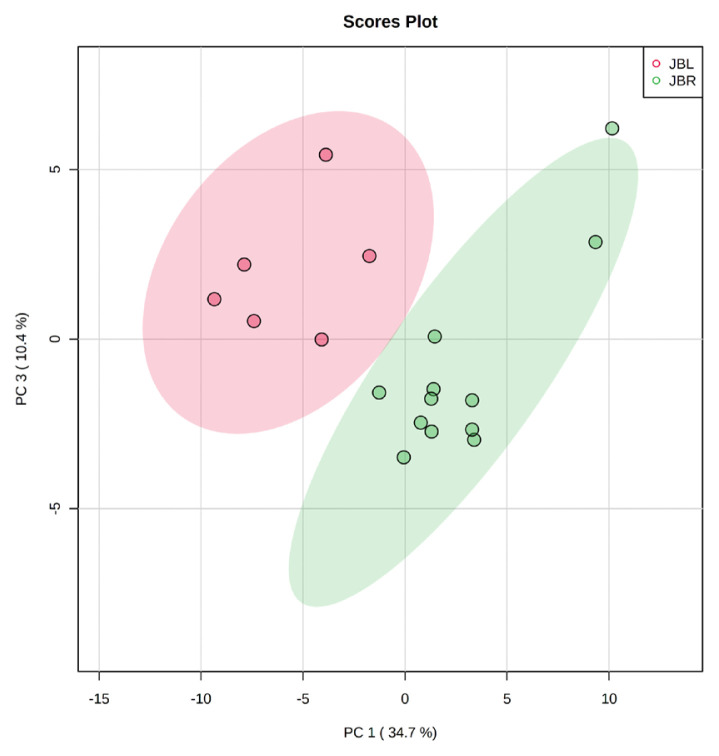
Results of principal component analysis (PCA) for metabolome profiles of postmortem Japanese Black (JBL, red) and Japanese Brown (JBRT, green) beef samples at day 14 postmortem.

**Figure 10:**
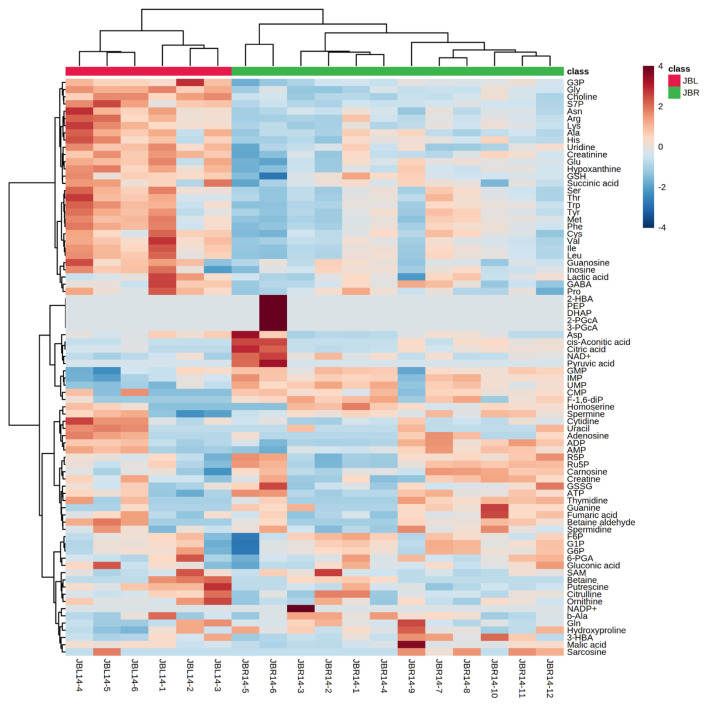
Heatmap result of hierarchical cluster analysis (HCA) of Japanese Black (JBL) and Japanese Brown (JBRT) beef samples at day 14 postmortem. The six JBL (red) and 12 JBRT (green) samples indicated under the heatmap were allocated to each breed group. The row displays the metabolite, and the column represents the sample. Metabolites with relatively low and high levels are displayed in light blue and brown, respectively. The brightness of each color corresponds to the magnitude of the difference when compared to the average value.

**Figure 11:**
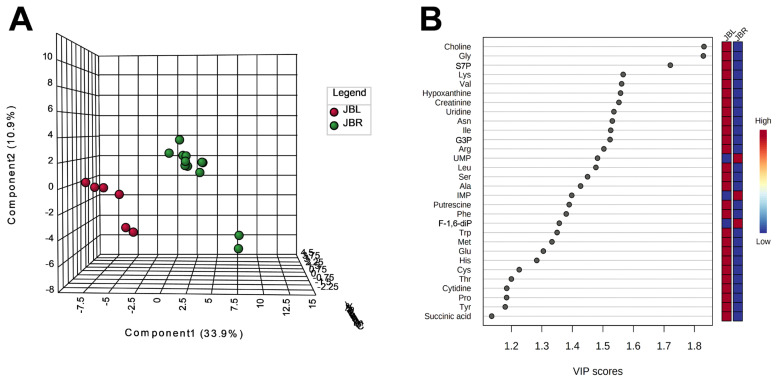
Result of partial least squares-discriminant analysis (PLS-DA) for metabolomic difference between Japanese Black and Japanese Brown beef at day 14 postmortem. (A) Score plot of component-1 and -2. (B) Variable importance in projection (VIP) scores of top 30 metabolites.

We further focused on the differences in aged beef between the breeds and compared the 14 d samples of JBRT (n = 12) with those of JBL (n = 6) cattle. The statistical analyses revealed that, in JBRT beef, the relative levels of the metabolite uridine monophosphate (UMP), IMP, and fructose-1,6-diphosphate (F-1,6-diP) were 3.53-, 1.71-, and 3.52-fold higher, respectively, than those in JBL beef (FDR<0.01) (Figure 8). The levels of choline, glycine, and sedoheptulose 7-phosphate (S7P) were lower in JBRT beef than in JBL beef (FDR <0.001). Other metabolites, including guanosine monophosphate (GMP) and AAs, also showed significant differences between breeds (Figure 8A). Aged beef samples were classified based on interbreed metabolomic differences in PCA and HCA. In the PCA results, beef samples were clearly grouped into JBRT or JBL breeds by PC1 and PC3 (Figure 9). The HCA results indicated that compared to JBRT beef, JBL beef was enriched with AAs at 14 days postmortem, whereas compared to JBL beef, JBRT beef was enriched with degradation products of nucleotide triphosphates (NTPs), such as UMP and IMP (Figure 10). Individual variations in the animal samples were observed in homoserine, spermine, cytidine, adenine diphosphate (ADP), AMP, carnosine, creatinine, GSSG, guanine, fumaric acid, betaine, and other metabolites, even within JBRT or JBL beef (Figure 10). In addition, the samples of JBRT and JBL cattle were discriminated using PLS-DA based on component-1 and -2 (Figure 11A). The top 30 metabolites with VIP score >1.0 were identified, based on the PLS-DA score plot (*R*^2^ = 0.934, *Q*^2^ = 0.772) from the comparison between the JBRT and JBL beef (Figure 11B). Among the top 30 metabolites, three (UMP, IMP, and F-1,6-diP) were higher and 27 were lower in JBRT beef than in JBL beef.

**Figure 12:**
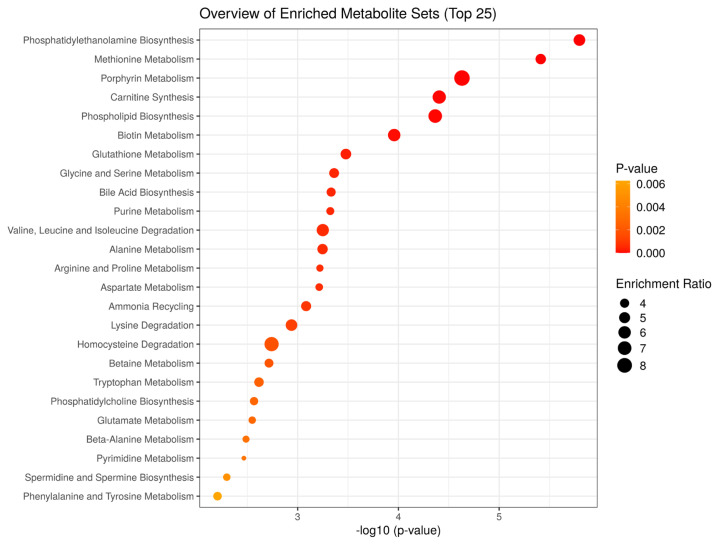
Result of metabolite set enrichment analysis for metabolomic difference between Japanese Black and Japanese Brown beef at day 14 postmortem. Enrichment ratio is computed by hits/Expected, where hits = observed hits; expected = expected hits.

To characterize the metabolic processes that differ between JBRT- and JBL-aged beef, we performed MSEA using the 110 metabolites in aged beef (day 14) between the JBRT and JBL breeds. The MSEA results showed that phosphatidylethanolamine biosynthesis, methionine metabolism, porphyrin metabolism, carnitine synthesis, phospholipid biosynthesis, biotin metabolism, glutathione metabolism, glycine and serine metabolism, bile acid biosynthesis, and purine metabolism were the top 10 different metabolism/metabolic pathways between beef of the two breeds (FDR<0.001) (Figure 12).

## DISCUSSION

In the present study, a total of 240 metabolites on HMD and/or KEGG database were observed across all the JBRT beef samples in CE-TOFMS metabolomics. These metabolome profiles are considerably abundant in water-soluble, metabolically functional metabolites that have hardly been observed so far. Owing to the large number of annotated metabolites, several pathways and metabolisms were newly extracted in the postmortem aging process of JBRT beef, such as pyruvate metabolism, acetyl group transfer into mitochondria, and mitochondrial β-oxidation. Furthermore, interbreed differences in aged beef metabolites and postmortem muscle metabolisms were determined between JBRT and JBL breeds.

In the LT muscle of JBRT cattle, changes in the levels of a variety of metabolites were observed during postmortem aging. As aging progressed, significant increases were observed in the levels of F6P, G6P, IMP, inosine, hypoxanthine, leucine, glutamate, isoleucine, nicotinamide, creatinine, and butyrylcarnitine. However, ATP, G3P, malic acid, NAD+, and GSSG levels decreased. Such changes accompanying aging were also apparent in NTPs, NDPs, F-1,6-diP, PRPP, and taurine which showed a decreasing trend, and CDP-choline, succinic acid, several AAs and peptides, thiamine, cytidine, uridine, 6-phosphogluconic acid (6-PG), ribose 5-phosphate (R5P), and ribulose 5-phosphate (Ru5P) which exhibited an increasing trend. These metabolites can be categorized into glycolytic products, purine compounds generated by ATP degradation, citrate cycle-related metabolites, protein degradation products, pentose phosphate pathway-related metabolites, and other metabolite groups. This suggests that metabolic pathways such as glycolysis, purine metabolism (especially ATP and GTP degradation), citrate cycle, protein degradation, and the pentose phosphate pathway progressed predominantly in the postmortem JBRT LT muscle. The AAs and peptides were likely generated by postmortem protein degradation rather than *de novo* biosynthesis or interconversion between AAs, as shown by accumulated evidence of protein degradation during postmortem aging [13,14,16,17].

The progress of these metabolic pathways was re-emphasized by the MSEA results, which demonstrated that pyrimidine metabolism, nicotinate and nicotinamide metabolism, purine metabolism, pyruvate metabolism, the Warburg effect, thiamine metabolism, citrate cycle, and pentose phosphate metabolism were the major metabolic pathways in postmortem JBRT beef (Figure 5). These metabolic pathways were also developed in postmortem JBL and porcine LT muscles [12,24,25]. The AA-associated metabolism extracted in MSEA, based on the accumulation of AAs and peptides, could be due to protein degradation, as observed in the postmortem aging of bovine muscles [12,24]. Postmortem protein degradation in porcine and bovine muscles is a well-established event based on many previous studies on troponin-T and other proteins [13,14,16,17]. Taken together, it is concluded that a series of coordinated metabolic events during the postmortem aging of JBRT beef is a conserved metabolic event in the postmortem skeletal muscle. In addition, the MSEA results of our study showed that fatty acid metabolism and mitochondrial metabolism were also activated in JBRT beef. Mitochondrial β-oxidation, fatty acid metabolism, and glycerolipid metabolism can also progress during the aging period, as shown in previous liquid chromatography-mass spectrometry metabolomics and lipidomics studies [26,33]. The present metabolomics results revealed an increasing trend of sugar phosphates (F6P, G6P, R5P, Ru5P), IMP, AAs including glutamate and creatinine, peptides, and 6-PG in postmortem JBRT beef aging. As sugars and AAs generate the Maillard reaction products through cooking process [10], these metabolite changes may contribute to flavor development in JBRT beef, together with increase in IMP, an important *umami* flavor compound. In this beef, redox metabolisms including glutathione and nicotinamide-related pathways seemed to change during the postmortem aging, which suggests progress of oxidation in the JBRT beef accompanying alteration of mitochondrial metabolisms. This may lead to lipid oxidation and discoloration of beef.

In the present study, we annotated 240 metabolites, the number of which was greater in JBRT beef in the present study than in JBL beef (171 metabolites) in a previous study [24]. This high annotation of metabolites in JBRT beef was largely due to the improved detection systems and metabolomic information in the database, compared to that of the previous study. Owing to this improvement, the bioinformatic analysis of JBRT beef using MSEA resulted in new findings on postmortem muscle metabolism, such as transfer of acetyl groups into mitochondria, riboflavin metabolism, mitochondrial electron transport chain, fatty acid metabolism, mitochondrial β-oxidation of long/medium chain saturated fatty acids, butyrate metabolism, and inositol (phosphate) metabolism (Figure 5).

We further analyzed the potential interbreed differences in the metabolome profiles of JBRT and JBL beef, based on the absolutely quantified metabolite levels to avoid great case-sensitive variation in the metabolomics analysis. In the HCA of samples during postmortem aging, interbreed differences were partly observed in the temporal changes in AAs, NTP degradation products, polyamines, glycolytic products, and other metabolites (Figure 6). However, in the interbreed comparison of D14 samples, a large variation in individual animal samples was observed in most of these metabolites, such as homoserine, spermine, ADP, AMP, carnosine, creatinine, GSSG, fumaric acid, and betaine in JBRT or JBL beef (Figure 10). Nevertheless, the levels of GMP, IMP, UMP, and F-1,6-diP were greater in JBRT beef than in JBL beef at day 14 postmortem, whereas the levels of choline, S7P, G3P, glycine, and other AAs were lower (Figure 8). Furthermore, according to PLS-DA results, JBRT and JBL beef samples could be discriminated by a set of these metabolites with high VIP scores. Accordingly, these metabolites could be useful as biomarkers for discriminating JBRT beef from JBL beef. Similarly, interbreed differences in muscle metabolites have been reported in bovine, porcine, and sheep muscles [30], some of which can be associated with differences in meat quality. In particular, IMP, phosphorylated sugars, and AAs contribute to meat flavor in beef and pork [34]. The differences in the metabolites between JBRT and JBL beef are likely associated with the differences in the flavor of each beef. Intriguingly, pH values at day 1 and 14 postmortem were different between the breeds in this study. This is likely associated with lower lactate concentration in JBRT beef than in JBL beef at day 1 and 14 postmortem, suggesting lower glycolytic activity or lower glycogen content in JBRT. It is well established that the ultimate pH value affects meat quality such as IMP content [12]. This may lead to higher umami flavor in JBRT beef than in JBL beef.

In addition, metabolites with interbreed differences can be generated through differently activated metabolism. MSEA in our study indicated that aged beef from the two breeds are different in metabolism related to not only various AAs but also phosphatidylethanolamine, phospholipid, glutathione, carnitine, purine, and phosphatidylcholine (Figure 12). This difference in purine metabolism could likely contribute to the higher accumulation of IMP in JBRT beef than in JBL beef. The lower accumulation of AAs in JBRT beef might be due to the lower activities of postmortem proteolysis than in JBL beef. Furthermore, the interbreed difference in carnitine metabolism may indicate that the beef of the two breeds differed in mitochondrial metabolism associated with fatty acid transportation and oxidation, which could further affect the redox state and glutathione levels in myofiber cells. The different fatty acid metabolic processes may also be linked to differences in intramuscular fat accumulation between the breeds. These differences may contribute to interbreed difference in flavor, tenderness, and meat color. Further investigation by sensory evaluation is required to understand the interbreed differences in meat quality between JBRT and JBL breed. Currently, little is known about the genetics, genomics, mRNA, and protein expression in JBRT skeletal muscle. Further studies are required to investigate mechanism underlying interbreed differences by analyzing the genetic background and effect of postmortem aging conditions on JBRT beef.

## CONCLUSION

In the LT muscle of JBRT cattle, metabolism related to purine/pyrimidine, nicotinate and nicotinamide, pyruvate, thiamine, amino sugar, fatty acid, butyrate, inositol, glycerolipid and mitochondrial fatty acid; and the citric acid cycle and pentose phosphate pathway were prominent pathways during the postmortem aging process. Of these, glycolysis, AA/peptide generation, and metabolism related to purine/pyrimidine, nicotinate/nicotinamide, pyruvate, and thiamine were the most common between JBRT and JBL breeds. Accumulation of IMP, UMP, and F-1,6-diP was greater, but that of choline, S7P, G3P, and various AAs was lower in aged JBRT beef than in JBL beef, indicating usefulness of these metabolites as potential biomarker candidates for discriminating between JBRT and JBL beef. The interbreed difference in beef metabolites could be due to metabolic differences related to AAs, phosphatidylethanolamine, phospholipid, glutathione, carnitine, purine, and phosphatidylcholine.

## Figures and Tables

**Table 1:** Loadings for PC1 and PC2 in the principal component analysis

Metabolite in PC1	Loading	Metabolite in PC2	Loading
	
1,5-Gluconolactone	0.1036	Carnitine	0.1500
Inosine	0.1021	N6-Methyllysine	0.1424
Cytidine	0.1021	SDMA	0.1417
Xanthine	0.1020	Betaine	0.1411
Uridine	0.1017	Citrulline	0.1372
3-Hydroxy-2-methyl-4-pyrone	0.1016	b-Ala-Lys	0.1354
NAcGlcNP	0.1016	Methylhistidine	0.1341
Gluconic acid	0.1011	Saccharopine	0.1326
S7P	0.1010	Theobromine	0.1323
Thiamine	0.1005	S-Methylmethionine	0.1321
Malic acid	−0.0791	2-Hydroxy-4-methylvaleric acid	−0.0650
S-Lactoylglutathione	−0.0792	4-Amino-3-hydroxybutyric acid	−0.0658
GSSG	−0.0814	Methylhistamine	−0.0697
ATP	−0.0815	Actinine	−0.0755
UTP	−0.0824	Diethanolamine	−0.0766
PRPP	−0.0836	N-Nitrosodiethanolamine	−0.0774
GTP	−0.0846	Isethionic acid	−0.0874
NADP+	−0.0891	Taurine	−0.0917
G3P	−0.0918	g-Glu-His	−0.1174
NAD+	−0.0954	Kynurenine	−0.1275

PC, principal component; SDMA, symmetric dimethylarginine; GSSG, oxidized glutathione; ATP, adenosine triphosphate; UTP, uridine triphosphate; PRPP, phosphoribosyl diphosphate; GTP, guanosine triphosphate; NADP+, nicotinamide adenine dinucleotide phosphate; G3P, glycerol 3-phosphate; NAD+, nicotinamide adenine dinucleotide.
